# Providing a gradient from low to high light intensity did not clearly affect the behavior, welfare or performance of either fast- or slower-growing broilers compared with a uniform low light intensity

**DOI:** 10.1016/j.psj.2026.107514

**Published:** 2026-07-28

**Authors:** Jerine A.J. van der Eijk, Henk Gunnink, Rodania Bekhit, Dennis E. te Beest, Ingrid C. de Jong

**Affiliations:** a Wageningen Livestock Research, Wageningen University and Research, PO Box 338, 6700 AH Wageningen, the Netherlands; b Biometris, Wageningen University and Research, PO Box 16, 6700 AA Wageningen, the Netherlands

**Keywords:** Broiler, Light intensity, Welfare, Behavior, Breed

## Abstract

One of the most important senses in chickens is vision and light (intensity, spectrum, etc.) influences broiler behavior, welfare and performance. However, a lot remains unknown about offering a choice in light intensity and how this affects behavior, welfare and performance, especially for slower-growing broilers which are increasingly used in broiler production systems. Therefore, we studied the effect of control (uniform 20 lux) versus gradient (range from 20 (dim) to 200 (bright) lux) lighting on behavior, welfare and performance of fast- and slower-growing broilers. Day-old chicks from a fast-growing (Ross 308) and slower-growing (Hubbard JA757) breed were housed until an approximate weight of 2.6 kg (33 kg/m^2^) in groups of 346 per pen (27.3 m^2^) with 8 pens per breed x light treatment combination. We assessed effects on behavior, responses to behavioral tests, activity, distribution, litter quality, welfare scores and performance. Slower-growing broilers had lower (better) scores for cleanliness compared to fast-growing broilers at 2.6 kg, but only in the gradient lighting. Slower-growing broilers showed more activity in bright lighting while activity of fast-growing broilers was not affected by light treatment. More fast-growing broilers were present in bright lighting while more slower-growing broilers were present in dim lighting. Thus, fast- and slower-growing broilers did not differ greatly in their responses to gradient lighting, with only minor differences in cleanliness, activity and distribution. Regardless of breed, gradient lighting reduced body weight and feed intake, and had no clear effects on welfare scores or litter quality compared to control lighting. Furthermore, gradient lighting reduced drinking behavior and increased fear of humans and play behavior (specific weights) compared to control lighting. Dim lighting reduced activity close to the wall and in gradient lighting, broilers seemed to be less uniformly distributed over the zones studied than in control lighting. Thus, offering broilers a choice in light intensity from low to high had no clear negative or positive effects on behavior, welfare or performance compared with a uniform low light intensity.

## Introduction

In chickens one of the most important senses is vision and light aspects (intensity, spectrum, etc.) can influence broiler behavior, welfare and performance (Meluzzi and Sirri, 2009; Wu et al., 2022). However, there are still many questions on how to provide light to broiler chickens, including light intensity. Broilers housed at low intensities (< 7 lux) were generally less active, performed less preening, foraging and eating and spend more time resting compared to those housed at high intensities (10-200 lux) (Newberry et al., 1988; Davis et al., 1999; Kristensen et al., 2006; Alvino et al., 2009a; Alvino et al., 2009b; Blatchford et al., 2009; Blatchford et al., 2012; Deep et al., 2012; Rault et al., 2017; Aldridge et al., 2022). Light intensity did not affect gait score or latency to lie (Newberry et al., 1988; Blatchford et al., 2009; Deep et al., 2010, 2013; Rault et al., 2017), but footpad lesions showed a linear increase with lowering light intensities from 10 to 0.5 lux (Deep et al., 2013) or 40 to 1 lux (Deep et al., 2010). In general, light intensity did not affect performance when comparing low light intensity (< 7 lux) to high light intensity (10-180 lux) (Newberry et al., 1988; Deep et al., 2010; Olanrewaju et al., 2014; Rault et al., 2017; Aldridge et al., 2022; Kim et al., 2022), but some studies reported either improved (5 versus 20 lux) (Rault et al., 2017; Aldridge et al., 2022) or reduced performance with lower light intensities (0.1-0.5 versus 1, 5 and 10 lux) (Deep et al., 2013). These findings suggest light intensity affects behavior and might also influence welfare and performance, although results are not always consistent. However, a lot remains unknown on how to optimize light intensity conditions for broilers.

Given a choice, broilers could be expected to choose a light intensity that is most suitable for their biological needs (Fraser and Nicol, 2018). Previous studies showed that broiler preferences depend on age, with broilers generally preferring higher light intensities when young and lower light intensities when older (Davis et al., 1999; Aldridge et al., 2022; van der Eijk et al., 2025), although this was not always found (Raccoursier et al., 2019). In addition, preferences may depend on the time of day, as more broilers were found in higher light intensities at the start and end of the light period (Raccoursier et al., 2019; van der Eijk et al., 2025). Furthermore, preferences also seem to depend on breed, with slower-growing broilers preferring the lowest intensity more compared to fast-growing broilers at older ages (van der Eijk et al., 2025). Moreover, in our previous study we noticed that there were always broilers present at each light intensity (choice between 0.2, 20, 50 or 1000 lux), suggesting individual preferences (van der Eijk et al., 2025). Thus, these findings indicate that preferences do exist and that they might depend on factors like breed, age and the time of day, and offering a choice might be beneficial for broiler welfare.

Recent studies have identified effects of distribution of light intensity on broiler behavior, welfare and performance. These studies compared fast-growing broilers housed in uniform lighting (5, 10 or 20 lux) to those in variable lighting, with high light intensities above the feeders (40 or 84-133 lux) and low light intensities close to the wall (2-5 lux). Broilers housed in variable lighting made more dustbathing holes, were more physically active, had less footpad lesions and were culled less due to leg problems compared to those housed in uniform 5 and/or 20 lux lighting, although performance was not affected (Kang et al., 2023). Furthermore, variable lighting had little impact on performance, footpad dermatitis, hock and gait scores, although standing and preening behavior were affected at specific ages compared to uniform 10 lux lighting (Shynkaruk et al., 2024). These findings give first indications of the effect of variable lighting on behavior, welfare and performance. However, it should be noted that light intensities were entangled with location and resources in these studies and only fast-growing broilers were studied. Thus, studies to date have focused on identifying effects of variable lighting (i.e. light intensity resource specific) on behavior, welfare and performance of fast-growing broilers. Yet, gradient lighting (i.e. light intensity not resource specific) was not studied and slower-growing broilers are increasingly used in broiler production systems with higher welfare requirements, mainly in North-Western Europe (Vissers et al., 2019). In addition, fast- and slower-growing broilers differ in their behavior (Wallenbeck et al., 2016; Dixon, 2020; Rayner et al., 2020; Dawson et al., 2021; de Jong et al., 2021; Güz et al., 2021; van der Eijk et al., 2022a, 2022b) and in light intensity preferences (van der Eijk et al., 2025), which might cause them to respond differently to gradient lighting.

Therefore, the aim of this study was to identify the effect of control (uniform 20 lux) versus gradient (20-200 lux) intensity lighting on behavior, welfare and performance of fast- and slower-growing broilers. We hypothesized that broilers would perform more active, comfort and foraging behaviors in the gradient lighting, as previously broilers were generally more active and performed more preening and foraging at higher light intensities or when housed with variable light intensity. With regard to welfare and performance, we hypothesized that broilers housed in gradient lighting would have improved welfare scores, most likely footpad dermatitis, and that performance would be similar between light treatments. We further hypothesized that effects of gradient lighting on behavior and welfare would be stronger for slower-growing broilers compared to fast-growing broilers, as they are in general found to be more active (Wallenbeck et al., 2016; Dixon, 2020; Rayner et al., 2020; Dawson et al., 2021; de Jong et al., 2021; Güz et al., 2021; van der Eijk et al., 2022a, 2022b).

## Materials and methods

### Experimental design and ethical approval

The experiment had a 2 x 2 factorial design with two broiler breeds, one fast-growing (F, Ross 308) and one slower-growing breed (S, Hubbard JA757) that received two light treatments (control or gradient lighting). The experiment was carried out at Spelderholt, the experimental farm of Aviagen (Lelystad, The Netherlands). Experimental procedures were checked with the national legislation on animal experiments by the institutional Animal Welfare Body. As procedures were non-invasive, this study was not considered to be an animal experiment under the Law on Animal Experiments, as confirmed by the institutional Animal Welfare Body (3rd of January, 2023, Lelystad, The Netherlands).

#### Animals and housing

Day-old broiler chicks, originating from a parent stock of 37 weeks of age (F) or 40 weeks of age (S), were obtained from a commercial hatchery (Probroed & Sloot, Langenboom, The Netherlands). Both breeds were incubated in the same way, i.e., with light (± 275 lux) and access to feed during hatching. A total of 5,536 broilers per breed were randomly allocated to the 2 light treatments, resulting in 4 experimental groups. Each experimental group was replicated 8 times (8 pens), with a total of 32 pens (each of 27.3 m^2^). There were 8 rooms/blocks consisting of 2 rows with 2 pens per row. Light treatments were assigned to a specific room with 4 rooms per light treatment and breeds were pseudo-randomly assigned within a room in such a way that each breed appeared once per row. Broilers were housed in groups of 346 (12.7 birds/m^2^) with an exact 50/50 male/female distribution (i.e. straight run). Depopulation was targeted at an approximate weight of 2.6 kg (33 kg/m^2^), resulting in depopulation at 38 (F) and 56 days of age (S).

Rooms were climate controlled with 34 °C at arrival which gradually decreased to a constant temperature of 18 °C at 40 days of age. Floor pens (27.3 m^2^, length 6.75 and width 4.05 m) had wood shavings as litter and further included 6 pan feeders and 36 nipple drinkers with cups. Pen fences were extended using a net up to 2 m high to avoid (slower-growing) broilers from escaping to other pens. Broilers had *ad libitum* access to feed and water. A three-phase feeding schedule was applied where broilers received a starter diet (0-14 days), grower diet (15-29 days) and finisher diet (> 29 days). The starter diet was crumbled, while the other diets were pelleted (diameter 3 mm). Coccidiostats were added to the diet (Maxiban® for starter, Sacox® for grower and finisher). Fast- and slower-growing broilers received the same diet. All diets were produced and pelleted by ABZ Diervoeding (Eindhoven, The Netherlands). Chicks were vaccinated against Infectious Bronchitis (PoulvacIB QX), and coccidiosis (Paracox-5) and received a probiotic (Aviguard) at the hatchery. Chicks were further vaccinated for Newcastle Disease (Avinew NeO) at 10 days of age, for IB 4-91 at 14 days of age and for Gumboro (D78) at 21 days of age.

#### Light

Locations of light treatments were fixed throughout the experiment, but two different lay-outs were used for the gradient lighting (with 4 replicates per breed) to avoid that a preference for a certain light intensity was entwined with the location of that light intensity as explained below.

The lighting schedule used was 24L:0D at arrival, 23L:1D from day 1 to 2 and 18L:6D from day 3 onwards. A twilight period of 30 min when transitioning from light to dark and vice versa was used, with lights starting to turn on at 01:00 or 04:30 and turn off at 23:30 or 22:00 with lights completely off at 24:00 or 22:30. Lights were fully on between 01:30 and 24:00 or 05:00 and 22:00. Lights were installed approximately 2.6 m above the floor (NatureDynamics Dome for Broilers, Once by Signify, USA). In the gradient lighting, lights were installed either in line with the center isle of the room (20 lights in total, 2 rooms) or in line with the back wall of the room (11 lights per wall, 22 lights in total, 2 rooms). In the control lighting, lights were installed evenly across the room (16 lights in total, 4 rooms). Light intensity (lux) at chick height (±25 cm) was measured from 9 locations per pen (evenly across each pen) and 4 pens per treatment (two per breed) using a spectral irradiance meter SIM-2 plus (Metrue Inc., USA), see Table 1. The light spectrum provided in all pens was Jungle Green colored LED light, with peaks in the blue, green and red part of the spectrum (van der Sluis et al., 2025b).

**Table 1:** Light intensity (lux) measurements (average and range between brackets) for the light treatments (control and gradient) at the start (day 0) and at 14 and 21 days of age. For gradient treatment pens, light intensity was averaged over measurements in the high, intermediate or low intensity area.

Light treatment	Day 0	Day 14	Day 21
Control	21.5 (18-27)	19.3 (14-25)	18.1 (14-23)
Light gradient (high)	209.3 (190-240)	198.8 (170-220)	193.0 (150-215)
Light gradient (intermediate)	82.3 (42-118)	76.5 (42-120)	72.9 (39-110)
Light gradient (low)	26.3 (20-33)	26.8 (20-35)	24.5 (17-32)

#### Performance

Body weight (BW) at pen level was measured using pen weight (all chicks per pen were weighed) at placement and depopulation (38 days of age for F, 56 days of age for S), and when switching between feed phases (at 15, 30 (F and S) and 42 (only S) days of age) a sample of 100 birds were weighed per pen. Feed and water intake were measured continuously per pen. Feed and water intake and feed conversion ratio were determined for the total rearing period. Feed conversion ratio was corrected for body weight at the moment of death. Mortality and culls were noted daily at pen level.

#### Behavioral observations

Behavior was observed live at pen level using instantaneous scan sampling four times per breed at 7-8, 17-18, 24-25 and 35-36 days of age for F broilers and 9-10, 22-23, 30-31 and 45-46 days of age for S broilers. These ages were chosen based on similar target weights of F and S broilers (0.2, 0.7, 1.2 and 2.2 kg, respectively). Actual weights during observations slightly differed from target weights, see Table 2. From this point forward, average weights are used as target weights.

**Table 2:** Target weight (in kg), actual body weight (in kg) for both fast- (F) and slower-growing broilers (S) with days of age between brackets, the difference (in kg) and average body weight (in kg) at each target weight.

Target weight	0.2	0.7	1.2	1.9	2.2	2.5
F	0.30 (7-8)	0.83 (17-18)	1.45 (24-25)	2.13 (32)	2.43 (35-36)	2.56 (37)
S	0.26 (9-10)	0.78 (22-23)	1.25 (30-31)	2.13 (42)	2.33 (45-46)	2.63 (53)
Difference (F – S)	0.04	0.05	0.20	0.00	0.10	0.07
Average ((F + S)/2)	0.3	0.8	1.4	2.1	2.4	2.6

On each observation day specific pens were observed once in the morning (08:00 – 12:30) and once in the afternoon (13:30 – 17:00). Each observation consisted of scanning two fixed areas (± 7 m^2^) 4 times after a 5 min habituation period. The areas were two quarters of the pen one in the back and one in the front where bales were not placed. Per scan, the behavior of all broilers in the area was scored according to the ethogram in Table 3. Behavioral observations were performed by one trained observer.

**Table 3:** Ethogram used for behavioral observations adapted from source: (van der Eijk et al., 2025; van der Sluis et al., 2025b).

Behavior	Description
Eating	Having the head above or in the feeder or pecking at feed in the feeder
Drinking	Pecking at the drinking nipples or cup beneath the drinking nipple
Locomotion	Walking, running, jumping or hopping without performing any other type of behavior
Inactive	Sitting or lying while not engaged in any other activities
Standing	Standing without performing any other type of behavior
Ground pecking	Pecking at the ground or litter while sitting or lying
Foraging	Pecking and/or scratching at the ground or litter while standing
Comfort	Preening (manipulating one’s own feathers with the beak or paws), stretching, wing flaps, feather ruffles, shakes (outside the context of dust bathing)
Dustbathing	Rubs head and body against the ground, pecks and scratches while lying on the side, distributes substrate over body or shakes off substrates from feathers
Aggressive behavior	All elements of aggressive behavior, such as hopping oriented towards another chicken, threatening (both upright position), leaping, kicking, wing flapping or aggressive pecking (pecking directed to the head)
Other	All other behaviors not described above

#### Human approach test

Behavioral tests were performed after each pen observation during the morning sessions.

The observer walked to the same location in each pen (between the wall/fence and drinking line) in a normal pace, turned around and directly started the observations. The number of broilers within 0.5 m in front of the observer was recorded every 30 sec during 3 min. In addition, the latency of the first broiler to enter the circle of 0.5 m around the observer was recorded.

#### Novel object test

This test was performed directly after the human approach test. The observer presented a novel object to the broilers by placing it in the litter, raising slowly, walking backwards for appr. 1.5 m and directly started observations. The number of broilers within 0.3 m of the object was recorded every 30 sec during 3 min. In addition, the latency of the first broiler to enter the circle of 0.3 m around the object was recorded. The novel object differed for each target weight with the following order: a golf ball wrapped in aluminum foil (diameter 4.5 cm), a rubber duck (length 6.5, width 5, height 6 cm), a PVC block wrapped in colored tape (green, white, black, yellow and red) (length 5, width 2 and height 10 cm) and PVC tube wrapped in colored tape (green, white, black, yellow and red) (length 50, diameter 3.2 cm).

#### Free-space test

The free-space test was adapted from (Liu et al., 2020) to identify play behavior. This test was performed inside each pen directly after the novel object test. The observer walked from the front of the pen halfway towards the back of the pen, turned around and walked towards the front of the pen and tried to drive as much broilers away from the area between the feeder and drink line and wall/fence and drink line. The area was video recorded for 5 min using a camera on a tripod. From the recorded videos, the observer used continuous all-occurrences sampling of specific behaviors (Table 4) over the whole 5 min observation period.

**Table 4:** Ethogram used for the free-space test.

Behavior	Description
Running	Forward movement, often including rapid direction change, at least twice the normal walking speed. No wing flapping involved.
Frolicking	Forward movement, at least twice the normal walking speed, with wings extended to each side or flapping, often includes sudden direction change
Wing flapping	Rapid bilateral up and down movements of wings while standing still or normal walking speed. Excludes wing flaps performed by a bird to balance itself on the drinker or feeder line/weighing scale.
Sparring	Two birds interact face to face as in fighting. May include hopping or chest bumping but no physical contact necessary. Brief, with no aggressive pecking. Each interaction between two birds was counted once.

#### Welfare measurements

Welfare measurements were assessed twice per breed at 32 and 37 days of age (F), and at 42 and 53 days of age (S), these ages were chosen based on similar target weights of F and S broilers (1.9 and 2.5 kg, respectively). Actual weights during assessment slightly differed from target weights, see Table 2. From this point forward, average weights are used as target weights. Welfare measurements included gait score, footpad dermatitis (FPD), hock burn (HB), cleanliness and skin lesions of 15 males and 15 females per pen (n = 30 per pen) according to Welfare Quality protocol (Welfare Quality®, 2009). To assess the quality of locomotion, gait scores were recorded and each bird was assigned a score between 0 (perfect) and 5 (unable to walk). FPD and HB were assigned a score between 0 (no lesions) and 4 (severe lesions). Cleanliness was scored by inspection of the breast area and assigned a score between 0 (completely clean) and 3 (very dirty). Skin lesions were assigned a score 0 (no scratches or wounds), 1 (single scratch or small wound ≤ 0.5cm^2^) or 2 (multiple scratches and/or large wounds > 0.5cm^2^). All welfare measurements were performed by one trained observer.

#### Litter quality

Litter quality was assessed by one trained assessor in each pen on the same days as behavioral observations. Litter was scored on a scale of 1 to 10 for friability and wetness as previously described by (van Harn et al., 2009), where litter score 1 corresponded with low litter quality (very wet, completely caked) and score 10 corresponded with high litter quality (dry, completely friable).

#### Activity and distribution

For data collection of activity and distribution, cameras (Hikvision DS-2CE16 H5T-ITE with 2.8 mm lens) were mounted in the center of the wall at approximately 3 m height, providing a view of the complete width of the pen and half of the length of each pen. Recordings were made 2 days per week between 5:00 and 22:00. The video frames had a resolution of 1920 x 1080 pixels and were recorded at a framerate of 15 frames per second. Cameras were connected to recorders and videos were stored on external hard disks every two weeks and transferred to large-scale storage with daily back-up. Part of the recordings were lost due to storage errors. For each age and breed there was a total of 2 replicates (2 pens) for control lighting and 4 replicates (4 pens) for dim lighting. For bright lighting, the number of replicates varied per age with a maximum of 4 replicates (4 pens) and at specific ages (week 4 and 5) for the fast-growing breed only 1 pen was available for video analysis (see supplementary Figure S1 D, E and F).

**Fig. 1:**
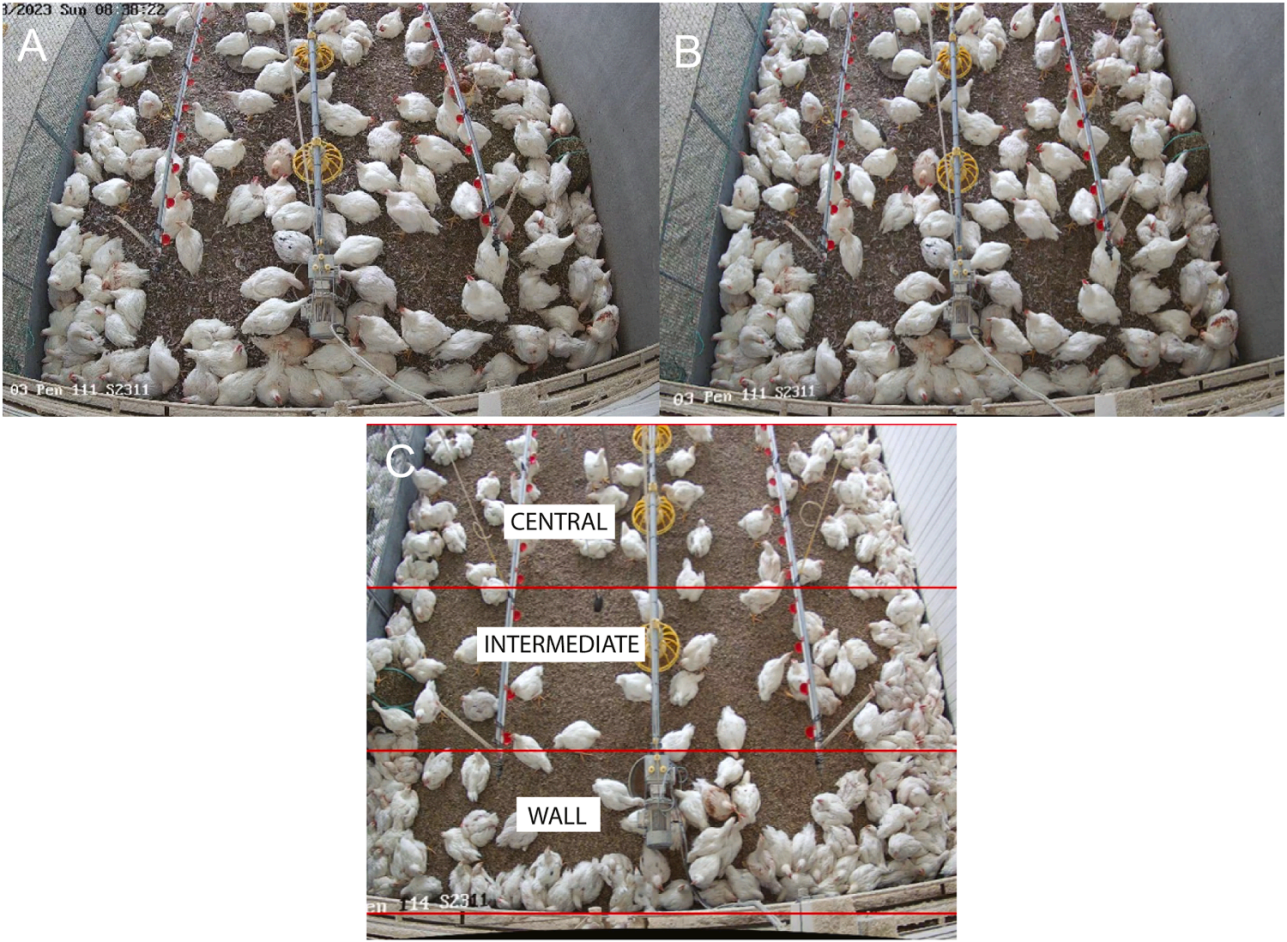
Comparison of the image before (A) and after (B) barrel distortion correction. C) Indication of the different zones (central, intermediate and wall) per pen.

The recordings suffered from barrel distortion, meaning that the outer regions of the image were compressed more than the inner ones (Chen et al., 2009). This caused straight lines to appear abnormally curved from the center of the image. To ensure accurate spatial analysis, this distortion was corrected prior to any zone-based segmentation of the pen. An undistortion transformation was applied using OpenCV (version 4.10.0), enabling a more uniform spatial representation across the frame. Fig. 1 illustrates the difference between the original distorted frame (A) and the corrected version (B). Following this correction, the pen was divided into three equal zones for subsequent analysis: near the back wall (wall zone), near the center (central zone) and the area in between the wall and center (intermediate zone) as indicated in Fig. 1C. These zones were chosen to identify effects of different light intensities of gradient lighting in relation to different resources (wall, feeders and drinkers) in comparison to control lighting on activity and distribution.

In order to count the number of broilers present in each zone an AI-based broiler detector model was used for detection, which was previously developed and described by (van der Sluis et al., 2025b). The detector was based on YOLOv8 (Jocher et al., 2023), trained on 3,002 images and validated on 503 images. The image dataset was collected from a diverse collection of video recordings representing various pen configurations. The dataset included 1,592 images from a prior bird preference study (van der Sluis et al., 2025b) and an additional 1,913 images sourced from videos recorded in this study and from (van der Sluis et al., 2025a). Annotation was performed using the LabelImg (version 1.8.6) tool (Tzutalin, 2015), with bounding boxes drawn around each individual bird. The model achieved an average precision (AP) of 96.2%, a recall of 93.7% and a F1-score of 94.9% at Intersection over Union (IoU) 0.5 and a mean AP of 73.0% across IoU thresholds from 0.5 to 0.95.

Broiler counts and their bounding boxes were detected at one-second intervals. The activity level within each zone was estimated using frame differencing, a common technique for motion detection. Motion detection refers to the process of identifying changes in the position of an object relative to its environment, or changes in the environment relative to a stationary object (Zhao and Pietikainen, 2007; Shafie et al., 2009). Pixel differences is one of the simplest methods for detecting motion in a video sequence (Rakibe and Patil, 2013; Singla, 2014; Wang et al., 2018). It works by comparing two consecutive frames in a video to find the differences in pixel values, which indicate movement within the time interval. However, some factors, such as changes in lighting conditions or the movement of feeder or drinker lines, can be falsely interpreted as motion within the pen. To minimize the influence of surrounding changes on detecting the activity level of the broilers, the frame difference was computed using binary masks, where pixels inside detected bounding boxes were assigned a value of 1 (white), and all other pixels were set to 0 (black). The absolute difference between consecutive binary masks was then calculated, and the number of changed pixels (CP) within each zone was used as a proxy for movement:

$$CP=\;\sum_{\left(x,\;y\right)\in P_{z}}^{n}\left|M_{t}\;\left(x,\;y\right)-\;M_{t-1}\;\left(x,\;y\right)\right|$$

Where *M_t_* (*x, y*) ϵ (0,1) is the binary mask value at pixel (*x, y*) at the time *t*, and *P_z_* represents the set of pixels within a zone.

For each zone, changed pixels (CP) was computed every second and normalised by the mean area of the detected bounding boxes in the current and previous seconds. Due to perspective distortion, zones closer to the camera appeared larger than those farther away, despite being physically equal in size. To correct for this, a zone-specific scaling factor (SF) was applied to adjust for the apparent area differences, ensuring that the activity metric better reflects true spatial occupancy.

The final relative activity level per zone was calculated as:

$$Relative\_CP_{z}=\;\frac{CP_{z}}{\;\left(\frac{\left(\sum bbx\_area_{t}+\sum bbx\_area_{t-1}\right)}{2}\right)\;}*SF_{z}$$

where:

• *CP_z_* is the number of changed pixels in zone *z*,
• ∑ *bbx_area_t_* + ∑ *bbx_area_t-_*_1_ are the total bounding box areas at time *t* and *t*-1, respectively,
• *SF_z_* is the scaling factor for zone *z*.

The relative CP values were aggregated over 5 min intervals (based on 300 one-second measurements) by computing their mean. A higher mean CP value indicated greater broiler activity within that time period.

#### Statistical analysis

All statistics were performed in R version 4.5.1. Performance data were analyzed for the total rearing period except for body weight which was analyzed per feeding phase. Linear mixed models were used consisting of a nested random effect for pen within room. For fixed effects, a full model was fitted first including treatment (control or gradient), breed, phase (only for body weight), all 2-way and the 3-way interactions (only for body weight) using the lme4 package.

Behavioral data were aggregated and expressed as percentage of broilers performing a certain behavior based on the total number of broilers observed. Behavioral test data were expressed as percentage of broilers performing a certain behavior based on the total number of broilers in the pen. Occurrences of aggressive and other behavior were very low and were therefore excluded from statistical analysis. For the human approach test, the number of birds approaching the human at 0.3 kg was very low and was therefore excluded from statistical analysis. Behavioral data and behavioral test count data were analyzed using generalized linear mixed models with a beta binomial distribution consisting of a nested random effect for pen within room. For fixed effects, a full model was fitted first including treatment (control or gradient), breed, target weight, all 2-way and the 3-way interactions using the glmmTMB package. Behavioral test latency data were analyzed using cox-proportional hazards regression mixed models consisting of a nested random effect for pen within room. For fixed effects, a full model was fitted first including treatment (control or gradient), breed, target weight, all 2-way and the 3-way interactions using the coxme package.

Activity and distribution data were analyzed using linear mixed models consisting of a nested random effect for pen within room. Activity data was square root transformed to obtain normality of residuals. Two types of models were used, one to identify effects between breeds over the first five weeks and one to identify effects within breeds (for fast-growing broilers over 5 weeks; for slower-growing broilers over 8 weeks). For the model with both breeds, fixed effects included treatment (control, bright or dim), breed, week, all 2-way and the 3-way interactions using the lme4 package. For the model within breeds, fixed effects included treatment (control, bright or dim), week and the 2-way interaction using the lme4 package.

For welfare and litter scores, a different statistical approach was used to assess the effects over multiple scores. Scores were analyzed with ordinal regression using R package ordinal. Some scores were aggregated before analysis (for gait, score 0 + 1, and 3 + 4, score 5 did not occur; for footpad dermatitis, score 2 + 3, score 4 did not occur; for hock burns, score 3 + 4; for cleanliness, score 3 did not occur, and for friability score 4 + 5). For friability, target weight 0.3 kg was excluded from analysis as all pens had score 10. For wetness, data was not analyzed as variability between pens was very low or non-existent for all target weights. The fitted models included a nested random intercept for room and pen and were fitted using the function clmm. For fixed effects, a full model was fitted first including treatment, breed, target weight, all 2-way and the 3-way interaction. The estimates were centered and shown on the logit scale used to fit the ordinal regression model.

For all analyses, except for activity and distribution data, backward selection was used, the interactions that were not significant were dropped (P-value > 0.05), except for treatment*breed which was always included. For the analyses of behavior, activity and distribution, per model term (main effects and interactions) and across the analyzed response variables (behaviors, activity or distribution in the three zones), P-values were adjusted for multiple comparisons using the Holm correction with the p.adjust function in R. For all analyses, differences between the different levels were determined via pair-wise contrasts using the emmeans package. Post hoc pairwise comparisons were corrected by Tukey-Kramer adjustment. We focused on significant differences within treatment, breed, phase, week or target weight.

## Results

### Performance

**Fig. 2:**
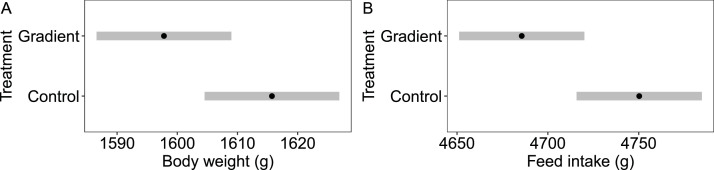
Model estimates of the mean (black dot) for A) body weight in grams or B) feed intake in grams with 95% confidence interval (grey area) for the gradient and control lighting treatments (*P <* 0.05).

See supplementary Table S1 for means and standard errors of overall performance measurements. No interaction effect between breed and treatment was found for any of the performance measurements. For body weight, a significant breed * phase effect was found (*P <* 0.001). Slower-growing broilers had a lower body weight in the starter and grower phase than fast-growing broilers, while the opposite was found in the finisher phase. Furthermore, main treatment effects were found on body weight (*P <* 0.05) and feed intake (*P <* 0.05), with broilers housed in the gradient lighting having a lower body weight (Fig. 2A) and lower feed intake compared to the control lighting (Fig. 2B).

#### Behavioral observations

**Fig. 3:**
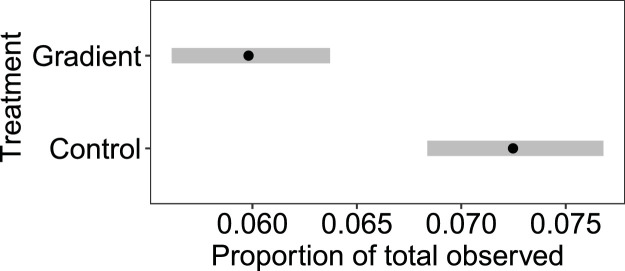
Model estimates of the mean (black dot) for drinking behavior expressed as a proportion from the total number of observed chickens with 95% confidence interval (grey area) for the gradient and control lighting treatments (*P <* 0.001).

See supplementary Table S2 for means and standard errors across target weights of behaviors scored. No interaction effects between breed and treatment, treatment and target weight or breed and target weight were found on behavior. Except for drinking behavior, where a trend for a treatment * target weight effect was found (*P <* 0.1) and for standing behavior, where a significant breed * target weight effect was found (*P <* 0.001). Broilers tended to drink less in the gradient versus control lighting at target weights of 0.8, 1.3 and 2.4 kg (data not shown). Slower-growing broilers were standing more than fast-growing broilers at all target weights (data not shown). A main treatment effect was found for drinking (*P <* 0.001), which was performed less in the gradient than in control lighting (Fig. 3). A trend for a main treatment effect was found for inactive ground pecking (*P <* 0.1), which tended to be performed more in the gradient versus control lighting (data not shown).

#### Behavioral tests

See supplementary Table S3 and S4 for means and standard errors across target weights of behavioral test responses scored. For latency to approach a human an interaction between breed * target weight was found (*P <* 0.001), with slower-growing broilers approaching a human faster compared to fast-growing broilers at 0.8 kg, while at 2.4 kg the opposite was found (data not shown). A trend for a main treatment effect was found for latency to approach a human (*P <* 0.1), with broilers tending to approach a human slower in the gradient versus control lighting (data not shown). For latency to approach a novel object an interaction between breed * target weight was found (*P <* 0.001), with slower-growing broilers approaching a novel object faster compared to fast-growing broilers at 0.3 kg (data not shown). A trend for a treatment * target weight effect was also found on latency to approach a novel object (*P <* 0.1), and after correction for multiple comparisons no significant differences were found between treatments within target weight.

**Fig. 4:**
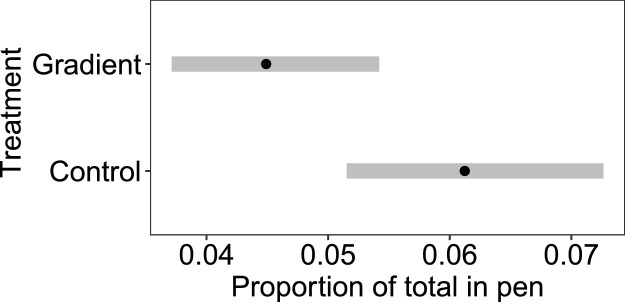
Model estimates of the mean (black dot) for chickens approaching the human expressed as a proportion from the total number of chickens in a pen with 95% confidence interval (grey area) for the gradient and control lighting treatments (*P <* 0.05).

For the number of broilers approaching a human a significant breed * target weight was found (*P <* 0.05). More slower-growing broilers approached a human at 0.8, 1.3 and 2.4 kg (data not shown). A main treatment effect was found for number of broilers approaching a human (*P <* 0.05), with less broilers approaching a human in the gradient versus control lighting (Fig. 4). No main effect of treatment or interactions between breed and treatment, treatment and target weight or breed and target weight were found on number of broilers approaching a novel object.

**Fig. 5:**
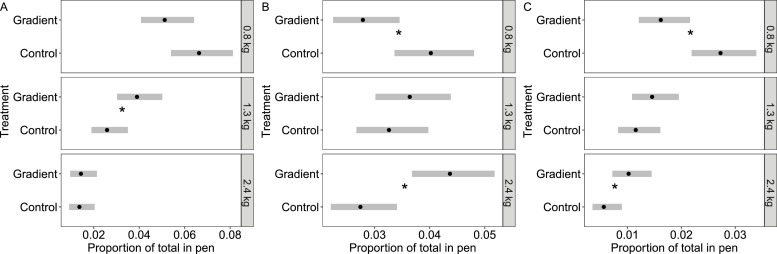
Model estimates of the mean (black dot) for A) frolicking, B) wing flapping and C) sparring behavior expressed as a proportion from the total number of chickens in a pen with 95% confidence interval (grey area) for the gradient and control lighting treatments per target weight. * indicate significant differences between treatments within target weight.

For frolicking a significant breed * target weight was found (*P <* 0.001), with more fast-growing broilers performing frolicking compared to slower-growing broilers at 0.8 and 2.4 kg (data not shown). For frolicking, wing flapping and sparring a significant treatment * target weight interaction was found (*P <* 0.05, *P <* 0.001, *P <* 0.01, respectively) (Fig. 5) and for running a trend for a treatment * target weight interaction was found (*P <* 0.1). More broilers tended to show running in the gradient versus control lighting at 2.4 kg (data not shown) and more broilers were frolicking in the gradient versus control lighting at 1.3 kg (Fig. 5A). More broilers were wing flapping and sparring in the control versus the gradient lighting at 0.8 kg, while at 2.4 kg the opposite was found (Fig. 5B and C, respectively).

#### Welfare measurements

**Fig. 6:**
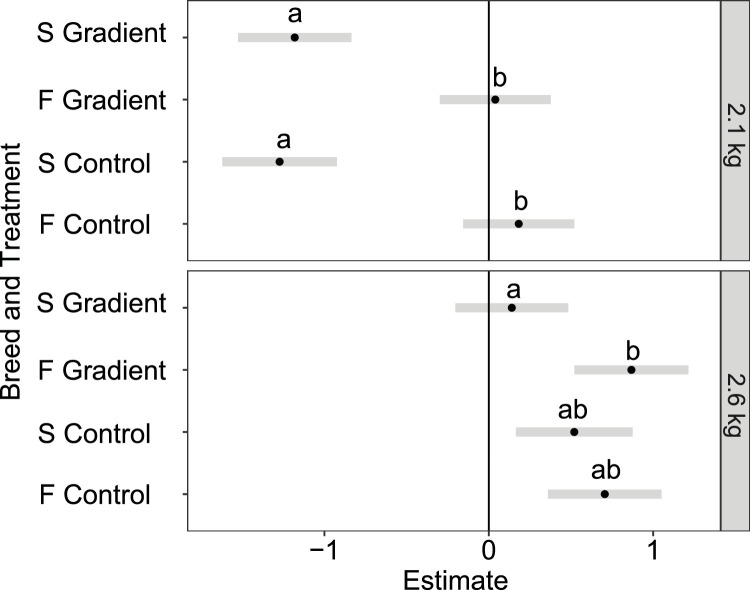
Mean estimates of the ordinal regression model (black dot) for cleanliness with 95% confidence interval (grey area) for the fast- and slower-growing breed (F = fast and S = slower-growing broilers) receiving either the control or gradient lighting treatment per target weight. Estimates are centered on zero per model. These estimates are on a log odds scale, higher values indicate higher odds of having a higher (ordinal) score. ^abc^ values lacking a common superscript differ significantly within target weight (*P <* 0.05).

See supplementary Tables S5a and S5b for means and standard errors of all welfare scores across target weights. An interaction between treatment, breed and target weight was found for cleanliness (*P <* 0.05). At 2.1 kg, slower-growing broilers had lower (better) cleanliness scores compared to fast-growing broilers regardless of treatment, while at 2.6 kg this was only found for broilers housed in the gradient lighting (Fig. 6). An interaction between breed and target weight was found for hock burns (*P <* 0.05). At 2.6 kg, slower-growing broilers had higher (worse) scores compared to fast-growing broilers, while this was not found at 2.1 kg (data not shown). For gait, footpad dermatitis and skin lesion scores no interactions were found, nor any main effects of treatment.

#### Litter quality

See supplementary Table S6 for means and standard errors of all litter scores across target weights. An interaction between breed and target weight was found for friability (*P <* 0.001). At 0.8, 1.3 and 2.4 kg, slower-growing broilers had higher (better) scores for friability compared to fast-growing broilers (data not shown). However, no interactions between treatment and target weight or breed and treatment were found nor a main effect of treatment. Only a trend between treatment and target weight was found for friability (*P <* 0.1), with gradient lighting pens tending to have lower (worse) scores for friability compared to control lighting pens at 0.8 kg (data not shown).

#### Activity and distribution

For activity and distribution, a different approach was used focusing on differences between light (gradient: dim, bright or control) instead of treatment (gradient or control) and per week instead of per target weight. In addition, the three zones were analyzed separately for both activity and distribution. For the wall and central zone, average light intensities are indicated in lux. For the intermediate zone, the range between the wall and central zone is indicated in lux. We identified effects when including both breeds (over breeds) and per breed (within breed). See supplementary Figure S1 for means of activity and Figure S2 for means of distribution where each pen is indicated by solid lines.

When comparing breeds over the first 5 weeks, the following effects were found.

**Fig. 7:**
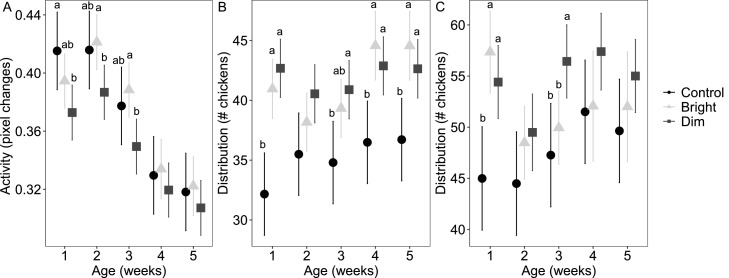
Model estimates of the mean (dot) for A) activity in the wall zone (control = 20 lux, bright = 204 lux and dim = 24 lux) expressed as pixel changes, B) distribution in the intermediate zone (control = 20 lux, bright = 97-204 lux and dim = 24-57 lux) and C) distribution in the wall zone (control = 20 lux, bright = 204 lux and dim = 24 lux) expressed as number of chickens with 95% confidence interval (lines) for the control lighting (black, circle), and for the bright (light grey, triangle) or dim (dark grey, square) lighting per week. ^ab^ values lacking a common superscript differ significantly within week (*P <* 0.05).

For activity in all three zones (central, intermediate and wall zones), interactions between breed and week were found (*P <* 0.001), where slow-growing broilers were more active compared to fast-growing broilers in week 2, 3, 4 and 5 (*P <* 0.01) (data not shown). Interactions between light and week were found for activity in the central zone (*P <* 0.01) and wall zone (*P <* 0.05). After correction for multiple comparisons no differences were found between lights within week nor a main light effect for activity in the central zone. For activity in the wall zone, broilers in dim lighting (24 lux) were less active compared to those in control lighting (20 lux) in week 1, and to those in bright lighting (204 lux) in week 2 and 3 (*P <* 0.05) (Fig. 7A). For activity in the intermediate zone, a main light effect was found (*P <* 0.05), but after correction for multiple comparisons no differences were found between lights.

**Fig. 8:**
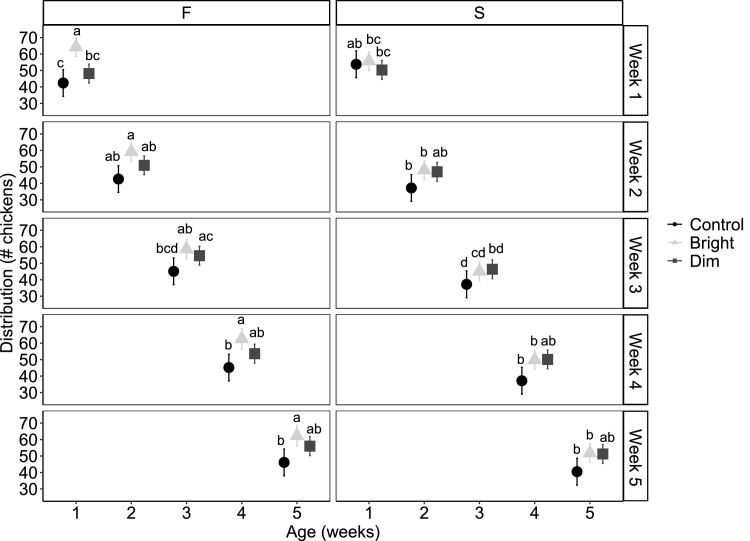
Model estimates of the mean (dot) for distribution in the central zone (control = 20 lux, bright = 97 lux and dim = 57 lux) expressed as number of chickens with 95% confidence interval (lines) for the control lighting (black, circle), and for the bright (light grey, triangle) or dim (dark grey, square) lighting and per week for both breeds (F = fast-growing broilers and S = slower-growing broilers). ^abcd^ values lacking a common superscript differ significantly within week (*P <* 0.05).

For the distribution in the central zone a three-way interaction was found between breed, week and light (*P <* 0.001) (Fig. 8). In week 1, more slow-growing broilers were in the central zone compared to fast-growing broilers in the control lighting (20 lux) (*P <* 0.01) while the opposite was found in the bright lighting. In week 2, 3, 4 and 5, less slow-growing broilers were in the central zone compared to fast-growing broilers in the bright lighting (97 lux) (*P <* 0.05). In week 3, less slow-growing broilers were in the central zone compared to fast-growing broilers in the dim lighting (57 lux) (*P <* 0.05). Interactions between light and week were found for distribution in the intermediate and wall zones (*P <* 0.05). For the distribution in the intermediate zone, less broilers were in control lighting (20 lux) compared to bright lighting (97-204 lux) in week 1, 4 and 5 (*P <* 0.01) and compared to dim lighting (24-57 lux) in week 1, 3, 4 and 5 (*P <* 0.05) (Fig. 7B). For the distribution in the wall zone, less broilers were in control lighting (20 lux) compared to bright (204 lux) or dim lighting (24 lux) in week 1 (*P <* 0.01 and *P <* 0.05, respectively). In week 3, less broilers were in dim lighting (24 lux) compared to bright (204 lux) or control lighting (20 lux) (*P <* 0.05) (Fig. 7C). For the distribution in the wall zone a significant interaction between breed and week was found (*P <* 0.001), with more slow-growing broiler being present compared to fast-growing broilers in week 1 (*P <* 0.001) (data not shown).

**Fig. 9:**
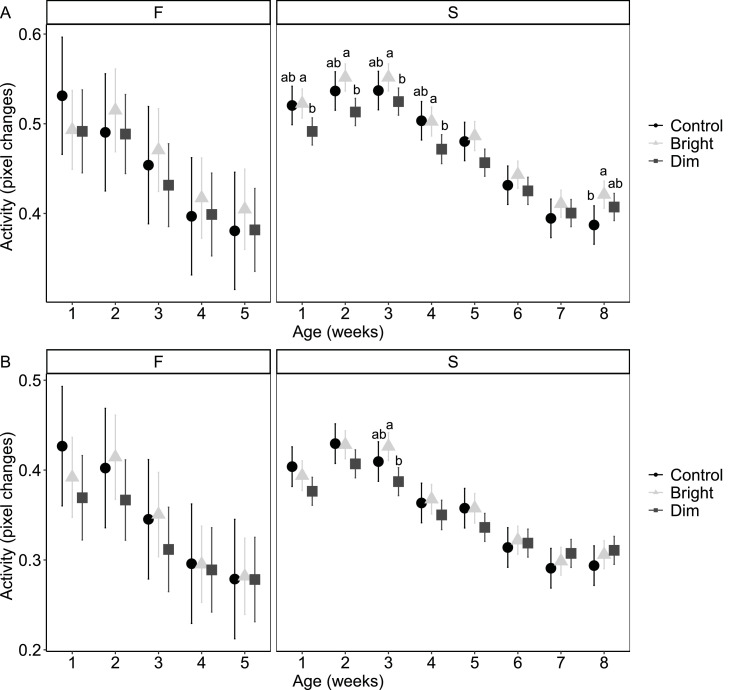
Model estimates of the mean (dot) for activity in the A) intermediate zone (control = 20 lux, bright = 97-204 lux and dim = 24-57 lux) and B) wall zone (control = 20 lux, bright = 204 lux and dim = 24 lux) expressed as pixel changes with 95% confidence interval (lines) for the control lighting (black, circle), and for the bright (light grey, triangle) or dim (dark grey, square) lighting per week and per breed (F = fast-growing broilers and S = slower-growing broilers). ^ab^ values lacking a common superscript differ significantly within week (*P <* 0.05).

**Fig. 10:**
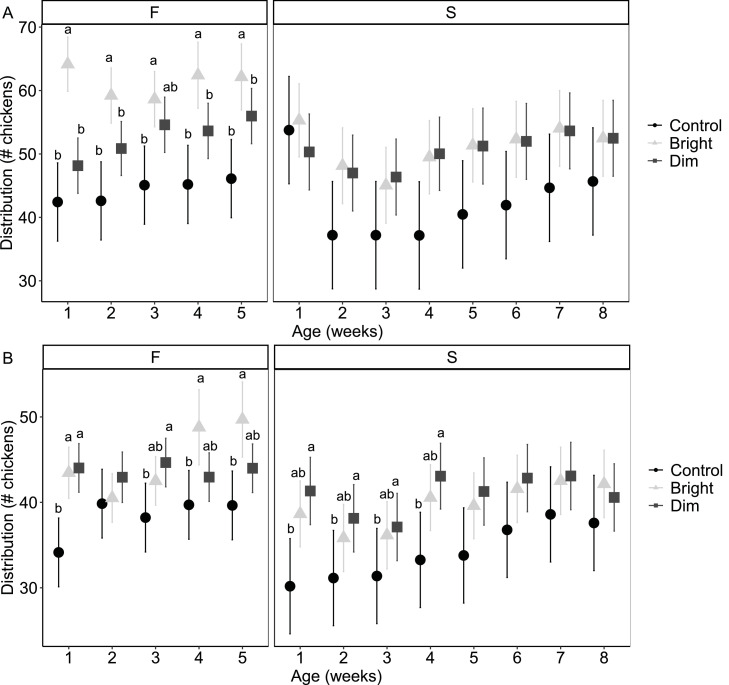
Model estimates of the mean (dot) for distribution in the A) central zone (control = 20 lux, bright = 97 lux and dim = 57 lux) and B) intermediate zone (control = 20 lux, bright = 97-204 lux and dim = 24-57 lux) expressed as pixel changes with 95% confidence interval (lines) for the control lighting (black, circle), and for the bright (light grey, triangle) or dim (dark grey, square) lighting per week and per breed (F = fast-growing broilers and S = slower-growing broilers). ^ab^ values lacking a common superscript differ significantly within week (*P <* 0.05).

For slower-growing broilers interactions between light and week were found for activity in the intermediate and wall zones, and distribution in the central and intermediate zones (*P <* 0.05). In the intermediate zone, slower-growing broilers were more active in the bright (97-204 lux) versus dim lighting (24-57 lux) in week 1, 2, 3 and 4, and versus control lighting (20 lux) in week 8 (*P <* 0.05) (Fig. 9A). In the wall zone, slower-growing broilers were more active in the bright (204 lux) versus dim lighting (24 lux) in week 3 (*P <* 0.01) (Fig. 9B). For the distribution in the central zone, no differences were found between lights after correction for multiple comparisons. For the distribution in the intermediate zone, more slower-growing broilers were in the dim versus control lighting in week 1 and 4 (*P <* 0.05) (Fig. 10B). No main light effects were found for activity in the central zone or distribution in the wall zone.

For fast-growing broilers interactions between light and week were found for activity in all zones (*P <* 0.05) and distribution in the central and intermediate zones (*P <* 0.001). For activity in all zones, no differences were found between lights after correction for multiple comparisons nor any main light effects. For the distribution in the central zone, more fast-growing broilers were in the bright (97 lux) versus control lighting (20 lux) in all weeks (*P <* 0.05), more were in the bright (97 lux) versus dim lighting (57 lux) in weeks 1, 2 and 4 (*P <* 0.05), and more were in the dim (57 lux) versus control lighting (20 lux) in week 5 (*P <* 0.05) (Fig. 10A). For the distribution in the intermediate zone, less fast-growing broilers were in the control (20 lux) versus bright lighting (97-204 lux) in weeks 1, 4 and 5 (*P <* 0.05), and in the control (20 lux) versus dim lighting (24-57 lux) in weeks 1 and 3 (*P <* 0.05) (Fig. 10B). For the distribution in the wall zone, a main light effect was found (*P <* 0.001), but after correction for multiple comparisons no differences were found between lights.

## Discussion

In this study, we identified the effect of control (uniform 20 lux) versus gradient (20-200 lux) intensity lighting on behavior, welfare and performance of fast- and slower-growing broilers. We found that gradient lighting reduced performance (body weight and feed intake) and had no clear effects on welfare scores or litter quality compared to control lighting. Gradient lighting affected some behaviors (reduced drinking) and increased fear of humans and play behavior (at higher body weights) compared to control lighting. Activity was reduced (young ages) in the dim lighting when compared to the control or bright lighting for the wall zone (both breeds) and the intermediate zone (slower-growing breed), suggesting bright lighting stimulated activity in the intermediate zone while dim lighting reduced activity close to the wall. With regard to distribution, more chicks were present in the bright lighting compared to the control or dim lighting for the central zone (fast-growing breed) and more chicks were present in the dim or bright lighting compared to the control lighting for the intermediate zone (both breeds), suggesting that broilers were less uniformly distributed over the zones studied in gradient compared to control lighting.

### Behavior, fear and play

Contrary to our hypotheses, we did not find differences in performance of active, comfort or foraging behaviors between light treatments. Broilers in gradient lighting showed less drinking behavior and tended to show more ground pecking behavior compared to those in control lighting. However, actual differences were relatively small (drinking 1.2%; ground pecking 0.3%). Previous studies mainly focused on comparing behavior in very low (< 7 lux) or high intensities (10-200 lux) (Newberry et al., 1988; Davis et al., 1999; Kristensen et al., 2006; Alvino et al., 2009a; Alvino et al., 2009b; Blatchford et al., 2009; Blatchford et al., 2012; Deep et al., 2012; Rault et al., 2017; Aldridge et al., 2022) and it might be that differences in behavior in our study were less clear because we compared uniform 20 lux versus gradient lighting (20-200 lux). Previously, broilers in variable lighting were more active and made more dustbathing holes (Kang et al., 2023), and they showed more standing and less preening behavior at specific ages (Shynkaruk et al., 2024), suggesting gradient lighting had limited effects on behavior. Less drinking behavior might be related to the lower feed intake and body weight found in gradient lighting, as there was a positive relationship between daily water and feed intake in poultry (Savory, 1978). Ground pecking while lying was included as foraging behavior in broilers as they reduced ground pecking while standing with increasing lameness (Weeks et al., 2000). Thus, a trend for more ground pecking might indicate increased performance of species specific natural behaviors in gradient lighting (Nicol et al., 2009), suggesting that offering a choice in light intensity supported the performance of natural behaviors. However, as differences were trending toward significance, further studies are required to confirm effects of gradient lighting on natural behaviors.

With regard to fearfulness, we found that less broilers approached a human and tended to approach slower in gradient compared to control lighting. These findings indicate higher fear of humans or lower motivation to explore (Forkman et al., 2007). However, actual differences were relatively small (1.4% and 4.6 sec). However, light treatments did not differ in responses to the novel object test, where fear and exploration motivation were also tested (Forkman et al., 2007). A previous study found that more broilers approached a novel object when housed in 5 lux uniform lighting compared to variable lighting (Kang et al., 2023), indicating they were less fearful or more motivated to explore. However, no differences were found between 20 lux uniform lighting and variable lighting, indicating differences found were not necessarily related to uniform or variable lighting (Kang et al., 2023). Recently, a study comparing artificial to natural lighting found that broilers in natural lighting, which had higher light intensity and was likely more variable, approached a novel object sooner and more (Youssef et al., 2026), indicating they were less fearful. Yet, no differences were found in response to a human or novel environment test (Youssef et al., 2026). Thus, findings regarding fear responses in broilers to gradient lighting are scarce and not always consistent.

To date, no study has identified effects of light intensity on play behavior. Here we found that boilers housed in gradient lighting showed more frolicking at 2.4 kg and tended to show more running at 1.3 kg. Furthermore, broilers in gradient lighting showed less wing flapping and sparring at 0.8 kg, and more at 2.4 kg compared to control lighting. However, actual differences were relatively small (1.5% for running, 0.2% for frolicking, 1.3 and 1.6% for wing flapping, and 1.0 and 0.5% for sparring). These findings suggest that gradient lighting reduced certain play behaviors at low body weight, and that it increased all play behaviors at higher body weights. Play is considered as a positive welfare indicator as it does not occur when there are fitness threats, is rewarding and increases the presence of opioid mediated positive affective states, it has immediate psychological and long-term fitness and health benefits, and is socially contagious (Held and Spinka, 2011). Thus, our findings might suggest that gradient lighting reduces positive welfare at low body weights, and that it increases positive welfare at higher body weights. However, we did not record spontaneous play behaviors, so we cannot conclude that broilers in gradient lighting played less in the test because they showed more spontaneous play at other times when young or that broilers in gradient lighting experienced a rebound effect because of fewer options due to a less uniform distribution at higher body weights. Further research is needed to identify effects of gradient lighting on other indicators of positive welfare, including spontaneous play behaviors.

Overall, our findings suggest that gradient lighting had minimal effect on behavior, increased fear of humans and play behavior at higher body weights. Breeds did not differ in their behavioral responses to control or gradient lighting.

### Activity and distribution

For activity and distribution a different approach was used, comparing light intensity in specific zones (gradient: dim, bright or control lighting) instead of treatment (gradient and control). No effects of light were found on activity in the central zone, this might be explained by lower differences in light intensity for the different light treatments in that zone (control 20 lux, bright 97 lux and dim 57 lux). In the intermediate zone, activity was higher for slower-growing broilers in bright (97-204 lux) compared to dim lighting (24-57 lux) in week 1-4, and compared to control lighting (20 lux) in week 8, suggesting higher activity in high light intensities as supported by previous studies (Newberry et al., 1988; Kristensen et al., 2006; Alvino et al., 2009a; Blatchford et al., 2009; Blatchford et al., 2012; Rault et al., 2017; van der Eijk et al., 2025). The higher activity might be related to more use of bales in higher light intensity areas, as a bale was located in the intermediate zone. Previous studies showed increased foraging in higher light intensity (Alvino et al., 2009a; Deep et al., 2012; van der Eijk et al., 2025) and more broilers gathered around bales when natural light (i.e. higher light intensity) was provided (Bailie et al., 2013). In the wall zone, activity was lower in dim lighting (24 lux) compared to bright (204 lux) or control lighting (20 lux) at young ages, and compared to bright lighting (204 lux) in week 3 for slower-growing broilers, further supporting that activity is higher in high light intensity areas. The lower activity might be related to more resting close to the wall and in lower light intensity areas, as chicks rested more close to objects/walls (Newberry and Shackleton, 1997; Cornetto and Estevez, 2001; Cornetto et al., 2002) and were less active in lower light intensities (Newberry et al., 1988; Kristensen et al., 2006; Alvino et al., 2009a; Blatchford et al., 2009; Blatchford et al., 2012; Rault et al., 2017; van der Eijk et al., 2025). The lower activity in dim (24 lux) versus control lighting (20 lux) in week 1 might be explained by young chicks choosing to rest more close to the wall in the dim versus control lighting. Chicks might move to the dim lighting close to the wall for resting as disturbances were more likely at the high light intensity on the opposite side of the pen because of increased activity levels (Newberry et al., 1988; Kristensen et al., 2006; Deep et al., 2010; Blatchford et al., 2012; van der Eijk et al., 2025). Control chicks that were resting and thus inactive, might be more evenly distributed across the pen walls as light intensity was similar throughout the pen. Thus, our findings suggest that bright lighting increased activity in the intermediate zone and that dim lighting reduced activity close to the wall.

To date, no study has identified effects of light intensity on the distribution of broilers. Here we showed that more fast-growing broilers were present in bright (97 lux) lighting compared to dim (57 lux) or control lighting (20 lux) (all ages), and in the dim (57 lux) compared to control lighting (20 lux) (week 5) in the central zone. For the intermediate zone, more broilers were in dim (24-57 lux) or bright lighting (97-204 lux) compared to control lighting (20 lux) (all ages). Within breed, more slower-growing broilers were in the dim (24-57 lux) versus control lighting (20 lux) (young ages) and more fast-growing broilers were in the dim (24-57 lux) or bright (97-204 lux) versus control lighting (20 lux) (all ages). These findings indicate that for both the central and intermediate zone, more broilers were present in higher light intensities. This might be related to feeding and drinking lines being included in both zones as previous studies showed that broilers perform more eating and drinking in high light intensities (Blatchford et al., 2012; Deep et al., 2013; van der Eijk et al., 2025), suggesting a preference for high light intensities when eating and drinking. Furthermore, in the intermediate zone a bale was included and more broilers gathered around bales when natural light (i.e. higher light intensity) was provided (Bailie et al., 2013), suggesting a preference for high light intensities when using bales. These findings might further indicate that broilers were less evenly distributed over the zones in gradient compared to control lighting. However, it should be noted that we did not have a complete overview of the whole pen. Further research should focus on spatial distribution in the whole pen or house in relation to uniform or gradient lighting. Still, the number of birds in control lighting, regardless of the zone analyzed, was often lower compared to the number of birds in gradient lighting. This is supported by previous findings in preference studies where birds preferred higher light intensities when young and lower light intensities when older (Davis et al., 1999; Aldridge et al., 2022; van der Eijk et al., 2025), although this was not always found (Raccoursier et al., 2019). For the wall zone, findings were less clear with more chicks being in the dim (24 lux) or bright (204 lux) versus control lighting (20 lux) in week 1, and more chicks were in the bright (204 lux) or control (20 lux) versus dim lighting (24 lux) in week 3. Young chicks might seek shelter close to the wall and might do this more in gradient versus control lighting, potentially because of increased activity and thus risk of disturbances in gradient lighting because of high light intensities (Newberry et al., 1988; Kristensen et al., 2006; Deep et al., 2010; Blatchford et al., 2012). Although it is unclear why more chicks were present in the bright or control lighting in comparison to dim lighting in week 3. Thus, our findings indicate that more broilers were present in the central and intermediate zone at higher light intensities, and for the wall zone findings were less clear. It further seems that broilers were less uniformly distributed across the zones in gradient lighting.

With regard to differences between breeds, within breed activity effects were only found for slower-growing broilers. This might indicate that slower-growing broilers were more responsive to differences in light intensity with regard to activity level compared to fast-growing broilers. Furthermore, more fast-growing chicks were present in the central zone compared to slower-growing chicks in the bright lighting (97 lux) (all ages), and in dim lighting (57 lux) (week 3), although the opposite was found in the control lighting (20 lux) (week 1). Fast-growing broilers might be more present in the central zone at higher light intensities compared to slower-growing broilers because they were found to eat and drink more (Dixon, 2020; Dawson et al., 2021; de Jong et al., 2021; van der Eijk et al., 2022a, 2022b). Furthermore, in a previous study we found that fast-growing broilers had a clearer preference for very high light intensities compared to slower-growing broilers (van der Eijk et al., 2025). Thus, breeds differed slightly in their responses to gradient lighting, where slower-growing broilers increased activity in bright lighting in the central zone and where more fast-growing broilers were present in bright lighting in the central zone.

However, it should be noted that the automated activity and distribution data had some limitations in that approximately half of the pen was recorded instead of the whole pen and that part of the data was missing due to errors in recordings. This calls for a follow up study where a complete view of the pen/house is observed and more replicates are included.

### Welfare and performance

Contrary to our hypotheses, gradient lighting did not improve welfare scores and performance was lower compared to control lighting. With regard to welfare scores, slower-growing broilers had lower (better) cleanliness scores compared to fast-growing broilers in the gradient lighting at 2.6 kg, while breeds did not differ in the control lighting. This finding suggests that breeds differed in their responses to gradient and control lighting with regard to cleanliness. However, no treatment effects were found on any of the other welfare indicators. This is supported by a previous study where variable lighting had little impact on welfare scores (FPD, HB and gait scores) compared to uniform lighting (Shynkaruk et al., 2024). Yet, another study found that broilers housed in variable lighting had less footpad lesions and were culled less due to leg problems compared to those in uniform lighting (Kang et al., 2023). Similarly, when comparing artificial to natural lighting, broilers in natural lighting had lower (better) FPD scores than those housed in artificial lighting, although no differences were found HB, gait scores or latency to lie (Youssef et al., 2026). In our study differences between gradient (dim or bright) and control lighting in activity level were limited to specific weeks (1 and 8), suggesting activity level did not differ greatly between treatments and this might explain why we found no differences in welfare findings. Our welfare findings were further supported by our findings for litter quality, where only a trend was found for friability being lower (worse) at a low body weight in the gradient lighting compared to control lighting. This finding would suggest worse welfare scores, but no differences were found at higher body weights regarding litter quality and there were no main treatment effects on any of the welfare scores. The minimal difference in litter quality might be explained by broiler distribution seeming to be less uniform in gradient (dim or bright) compared to control lighting as based on our distribution findings.

Broilers housed in gradient lighting had lower body weight (± 30 grams) and feed intake (± 64 grams) compared to control lighting. However, actual differences were relatively small and FCR was similar for both treatments. Our findings are supported by previous studies indicating variable lighting had little impact or did not affect performance compared to uniform lighting (Kang et al., 2023; Shynkaruk et al., 2024). Differences found were supported by behavioral findings, with gradient broilers performing less drinking behavior and eating and drinking being correlated, although no actual differences were found in eating behavior.

Overall, findings suggest that gradient lighting did not clearly affect welfare scores or litter quality and that performance was lower compared to control lighting. Breeds did differ in their response to control and gradient lighting with regard to cleanliness, but no differences were found for any of the other welfare indicators, litter quality or performance.

In conclusion, gradient lighting increased play behavior and fear of humans, and reduced performance and drinking behavior, with minimal effects on welfare scores or litter quality. Bright lighting stimulated activity in the intermediate zone while dim lighting reduced activity close to the wall. In gradient lighting, broilers were less uniformly distributed over the zones studied than in control lighting. Thus, offering broilers a choice in light intensity did not have clear negative or positive effects on behavior, welfare or performance in comparison to a uniform light intensity. Furthermore, fast- and slower-growing broilers did not differ greatly in their responses to gradient lighting, with only some differences in cleanliness, activity and distribution.

## Disclosures

The authors declare that they have no known competing financial interests or personal relationships that could have appeared to influence the work reported in this paper.

## Appendix Supplementary materials

Image, application 1

## Footnotes

Supplementary material associated with this article can be found, in the online version, at doi:10.1016/j.psj.2026.107514.
