# Supplementary material for: Providing a gradient from low to high light intensity did not clearly affect the behavior, welfare or performance of either fast- or slower-growing broilers compared with a uniform low light intensity
Source: Poult Sci. 2026 Jul 28;105(11):107514. doi: 10.1016/j.psj.2026.107514 (PMC13487386; doi:10.1016/j.psj.2026.107514)
Supplement: Supplementary file 1 [file mmc1.docx]

**Supplementary Material**

OFFERING BROILERS A CHOICE IN LIGHT INTENSITY

Providing a gradient from low to high light intensity did not clearly affect the behavior, welfare or performance of either fast- or slower-growing broilers compared with a uniform low light intensity

Jerine A.J. van der Eijk ^*,1^, Henk Gunnink^*^, Rodania Bekhit^*^, Dennis E. te Beest^#^, Ingrid C. de Jong^*^

^*^Wageningen Livestock Research, Wageningen University and Research, PO Box 338, 6700 AH Wageningen, The Netherlands

^#^Biometris, Wageningen University and Research, PO Box 16, 6700 AA Wageningen, The Netherlands

^1^Corresponding author: Jerine A.J. van der Eijk; Wageningen Livestock Research, Wageningen University and Research, PO Box 338, 6700 AH Wageningen, The Netherlands. E-mail: [jerine.vandereijk@wur.nl](mailto:jerine.vandereijk@wur.nl)

Supplementary Table S1. Effects of breed, treatment and their interaction on overall broiler performance. Values are expressed as means ± SE. F = fast-growing broilers (depopulation at 38 days of age); S = slower-growing broilers (depopulation at 56 days of age); C = Control; G = Gradient (n = 8 pens per breed * treatment combination).

|  |  | Body weight (g) |  | SE | Mortality % |  | SE | Feed intake (g) |  | SE | Water : Feed |  | SE | FCR |  | SE |
| --- | --- | --- | --- | --- | --- | --- | --- | --- | --- | --- | --- | --- | --- | --- | --- | --- |
| Breed | F | 2636^b^ | ± | 10,2 | 2,28^a^ | ± | 0,213 | 4099^b^ | ± | 13,8 | 1,76^a^ | ± | 0,005 | 1,50^b^ | ± | 0,002 |
|  | S | 2770^a^ | ± | 10,6 | 1,55^b^ | ± | 0,227 | 5337^a^ | ± | 22,2 | 1,65^b^ | ± | 0,005 | 1,86^a^ | ± | 0,002 |
| Treatment | C | 2718^a^ | ± | 21,2 | 2,15 | ± | 0,258 | 4750^a^ | ± | 163,8 | 1,70 | ± | 0,015 | 1,68 | ± | 0,047 |
|  | G | 2688^b^ | ± | 18,2 | 1,68 | ± | 0,200 | 4686^b^ | ± | 157,6 | 1,71 | ± | 0,014 | 1,68 | ± | 0,047 |
| Breed * Treatment | FC | 2647 | ± | 17,2 | 2,67 | ± | 0,373 | 4120 | ± | 20,1 | 1,75 | ± | 0,005 | 1,50 | ± | 0,003 |
|  | SC | 2789 | ± | 14,2 | 1,63 | ± | 0,262 | 5381 | ± | 33,0 | 1,64 | ± | 0,008 | 1,86 | ± | 0,004 |
|  | FG | 2625 | ± | 10,7 | 1,88 | ± | 0,095 | 4078 | ± | 16,9 | 1,76 | ± | 0,009 | 1,50 | ± | 0,001 |
|  | SG | 2751 | ± | 13,2 | 1,48 | ± | 0,388 | 5294 | ± | 22,2 | 1,66 | ± | 0,005 | 1,86 | ± | 0,002 |
| P-values | Breed | < 0.001 | | | 0.02 | | | < 0.001 | | | < 0.001 | | | < 0.001 | | |
|  | Treatment | 0.02 | | | 0.13 | | | 0.01 | | | 0.19 | | | 0.56 | | |
|  | Breed * Treatment | 0.74 | | | 0.29 | | | 0.36 | | | 0.31 | | | 0.85 | | |

^a-b^ Per factor (breed, treatment or interaction), values in a column lacking a common superscript differ significantly (P < 0.05).

Supplementary Table S2. Effects of breed, treatment and their interaction on broiler behaviour across target weights (0.3, 0.8, 1.3 and 2.4 kg). Values are expressed as means ± SE. F = fast-growing broilers; S = slower-growing broilers; C = Control; G = Gradient (n = 8 pens per breed * treatment combination).

|  |  | Eating |  | SE | Drinking |  | SE | Locomotion |  | SE | Standing |  | SE | Inactive |  | SE |
| --- | --- | --- | --- | --- | --- | --- | --- | --- | --- | --- | --- | --- | --- | --- | --- | --- |
| Breed | F | 6,5^a^ | ± | 0,28 | 7,6^a^ | ± | 0,22 | 2,2^b^ | ± | 0,09 | 1,4^b^ | ± | 0,07 | 70,9^a^ | ± | 0,50 |
|  | S | 5,7^b^ | ± | 0,26 | 5,7^b^ | ± | 0,17 | 3,9^a^ | ± | 0,14 | 6,2^a^ | ± | 0,34 | 65,7^b^ | ± | 0,56 |
| Treatment | C | 6,2 | ± | 0,26 | 7,3^a^ | ± | 0,21 | 3,0 | ± | 0,14 | 3,8 | ± | 0,39 | 68,1 | ± | 0,58 |
|  | G | 6,0 | ± | 0,29 | 6,1^b^ | ± | 0,22 | 3,1 | ± | 0,17 | 3,8 | ± | 0,39 | 68,5 | ± | 0,65 |
| Breed * Treatment | FC | 6,7 | ± | 0,39 | 8,3 | ± | 0,27 | 2,2 | ± | 0,11 | 1,4 | ± | 0,10 | 70,7 | ± | 0,69 |
|  | SC | 5,7 | ± | 0,34 | 6,3 | ± | 0,22 | 3,9 | ± | 0,17 | 6,3 | ± | 0,49 | 65,5 | ± | 0,69 |
|  | FG | 6,3 | ± | 0,42 | 7,0 | ± | 0,31 | 2,3 | ± | 0,16 | 1,5 | ± | 0,11 | 71,0 | ± | 0,73 |
|  | SG | 5,8 | ± | 0,41 | 5,1 | ± | 0,22 | 3,9 | ± | 0,23 | 6,2 | ± | 0,50 | 65,9 | ± | 0,88 |
| P-values | Breed | 0.02 | | | < 0.001 | | | < 0.001 | | | < 0.001 | | | < 0.001 | | |
|  | Treatment | 1.00 | | | < 0.001 | | | 1.00 | | | 1.00 | | | 1.00 | | |
|  | Breed * Treatment | 1.00 | | | 1.00 | | | 1.00 | | | 1.00 | | | 1.00 | | |

|  |  | Ground pecking |  | SE | Foraging |  | SE | Comfort |  | SE | Dust bathing |  | SE |
| --- | --- | --- | --- | --- | --- | --- | --- | --- | --- | --- | --- | --- | --- |
| Breed | F | 3,5 | ± | 0,14 | 0,3^b^ | ± | 0,03 | 5,7 | ± | 0,11 | 1,4 | ± | 0,10 |
|  | S | 3,4 | ± | 0,12 | 1,2^a^ | ± | 0,08 | 6,1 | ± | 0,17 | 1,2 | ± | 0,18 |
| Treatment | C | 3,3 | ± | 0,14 | 0,7 | ± | 0,08 | 5,7 | ± | 0,15 | 1,2 | ± | 0,12 |
|  | G | 3,6 | ± | 0,12 | 0,8 | ± | 0,08 | 6,1 | ± | 0,13 | 1,4 | ± | 0,17 |
| Breed * Treatment | FC | 3,4 | ± | 0,22 | 0,3 | ± | 0,05 | 5,5 | ± | 0,14 | 1,3 | ± | 0,14 |
|  | SC | 3,3 | ± | 0,18 | 1,1 | ± | 0,10 | 5,9 | ± | 0,25 | 1,1 | ± | 0,21 |
|  | FG | 3,7 | ± | 0,18 | 0,4 | ± | 0,04 | 6,0 | ± | 0,15 | 1,4 | ± | 0,16 |
|  | SG | 3,6 | ± | 0,16 | 1,2 | ± | 0,12 | 6,2 | ± | 0,22 | 1,3 | ± | 0,30 |
| P-values | Breed | 0.40 | | | < 0.001 | | | 0.19 | | | 0.09 | | |
|  | Treatment | 0.09 | | | 1.00 | | | 0.28 | | | 1.00 | | |
|  | Breed * Treatment | 1.00 | | | 1.00 | | | 1.00 | | | 1.00 | | |

^a-b^ Per factor (breed, treatment or interaction), values in a column lacking a common superscript differ significantly (P < 0.05).

Supplementary Table S3. Effects of breed, treatment and their interaction on broiler behavioural test responses across target weights (0.3, 0.8, 1.3 and 2.4 kg). Values are expressed as mean latency or percentage of broilers approaching ± SE. F = fast-growing broilers; S = slower-growing broilers; C = Control; G = Gradient (n = 8 pens per breed * treatment combination).

|  |  | Human approach test | | | | | | Novel object test | | | | | |
| --- | --- | --- | --- | --- | --- | --- | --- | --- | --- | --- | --- | --- | --- |
|  |  | Latency (s) |  | SE | Approach (%) |  | SE | Latency (s) |  | SE | Approach (%) |  | SE |
| Breed | F | 78,0 | ± | 10,21 | 3,7^b^ | ± | 0,50 | 35,8 | ± | 7,56 | 3,2^b^ | ± | 0,40 |
|  | S | 56,6 | ± | 9,21 | 6,7^a^ | ± | 0,70 | 22,7 | ± | 4,30 | 6,4^a^ | ± | 0,69 |
| Treatment | C | 65,0 | ± | 9,82 | 5,9^a^ | ± | 0,66 | 31,0 | ± | 6,69 | 5,0 | ± | 0,59 |
|  | G | 69,6 | ± | 9,81 | 4,5^b^ | ± | 0,60 | 27,5 | ± | 5,67 | 4,6 | ± | 0,60 |
| Breed * Treatment | FC | 76,6 | ± | 14,96 | 4,5 | ± | 0,82 | 38,8 | ± | 11,68 | 3,4 | ± | 0,58 |
|  | SC | 53,5 | ± | 12,62 | 7,2 | ± | 0,99 | 23,1 | ± | 6,44 | 6,6 | ± | 0,96 |
|  | FG | 79,4 | ± | 14,15 | 2,8 | ± | 0,54 | 32,7 | ± | 9,76 | 3,1 | ± | 0,55 |
|  | SG | 59,8 | ± | 13,60 | 6,2 | ± | 1,00 | 22,3 | ± | 11,68 | 6,2 | ± | 1,00 |
| P-values | Breed | 0.71 | | | < 0.001 | | | 0.81 | | | < 0.001 | | |
|  | Treatment | 0.08 | | | 0.01 | | | 0.51 | | | 0.72 | | |
|  | Breed * Treatment | 0.34 | | | 0.25 | | | 0.47 | | | 0.99 | | |

^a-b^ Per factor (breed, treatment or interaction), values in a column lacking a common superscript differ significantly (P < 0.05).

Supplementary Table S4. Effects of breed, treatment and their interaction on broiler behavioural test responses across target weights (0.8, 1.3 and 2.4 kg). Values are expressed as mean percentage of broilers showing a behaviour ± SE. F = fast-growing broilers; S = slower-growing broilers; C = Control; G = Gradient (n = 8 pens per breed * treatment combination).

|  |  | Free space test | | | | | | | | | | | |
| --- | --- | --- | --- | --- | --- | --- | --- | --- | --- | --- | --- | --- | --- |
|  |  | Running |  | SE | Frolicking |  | SE | Wing flapping |  | SE | Sparring |  | SE |
| Breed | F | 8,7^b^ | ± | 1,06 | 4,7^a^ | ± | 0,55 | 3,3 | ± | 0,18 | 1,2^b^ | ± | 0,15 |
|  | S | 12,7^a^ | ± | 0,83 | 2,7^b^ | ± | 0,31 | 3,7 | ± | 0,23 | 1,8^a^ | ± | 0,18 |
| Treatment | C | 10,0 | ± | 0,82 | 3,7 | ± | 0,50 | 3,4 | ± | 0,22 | 1,5 | ± | 0,19 |
|  | G | 11,3 | ± | 1,14 | 3,7 | ± | 0,44 | 3,6 | ± | 0,20 | 1,4 | ± | 0,15 |
| Breed * Treatment | FC | 7,7 | ± | 0,75 | 4,9 | ± | 0,86 | 3,2 | ± | 0,26 | 1,3 | ± | 0,22 |
|  | SC | 12,4 | ± | 1,30 | 2,5 | ± | 0,42 | 3,5 | ± | 0,36 | 1,7 | ± | 0,30 |
|  | FG | 9,6 | ± | 2,00 | 4,6 | ± | 0,71 | 3,3 | ± | 0,26 | 1,0 | ± | 0,21 |
|  | SG | 13,0 | ± | 1,06 | 2,8 | ± | 0,46 | 3,8 | ± | 0,31 | 1,8 | ± | 0,20 |
| P-values | Breed | < 0.001 | | | < 0.001 | | | 0.21 | | | < 0.001 | | |
|  | Treatment | 0.45 | | | 0.92 | | | 0.36 | | | 0.80 | | |
|  | Breed * Treatment | 0.70 | | | 0.87 | | | 0.74 | | | 0.22 | | |

^a-b^ Per factor (breed, treatment or interaction), values in a column lacking a common superscript differ significantly (P < 0.05).

Supplementary Table S5a. Effects of breed, treatment and their interaction on broiler gait score, cleanliness and skin lesions across target weights (2.1 and 2.6 kg). Values are expressed as mean percentage of broilers having a specific score ± SE. For gait, where score 0 = walking perfectly and 5 = unable to walk; for cleanliness, where score 0 = completely clean and score 3 = very dirty; and for skin lesions where score 0 = no scratches or wounds and 2 = multiple scratches and/or large wounds. F = fast-growing broilers; S = slower-growing broilers; C = Control; G = Gradient (n = 8 pens per breed * treatment combination).

|  |  | Gait score | | | | | | | | | | | | Cleanliness | | | | | | | | | Skin lesions | | | | | | | | |
| --- | --- | --- | --- | --- | --- | --- | --- | --- | --- | --- | --- | --- | --- | --- | --- | --- | --- | --- | --- | --- | --- | --- | --- | --- | --- | --- | --- | --- | --- | --- | --- |
|  |  | 1 |  | SE | 2 |  | SE | 3 |  | SE | 4 |  | SE | 0 |  | SE | 1 |  | SE | 2 |  | SE | 0 |  | SE | 1 |  | SE | 2 |  | SE |
| Breed | F | 4,0^a^ | ± | 0,94 | 56,7 | ± | 56,7 | 39,3 | ± | 2,66 | 0,1 | ± | 0,10 | 21,5^a^ | ± | 1,88 | 74,5 | ± | 1,61 | 4,0 | ± | 0,87 | 81,2^a^ | ± | 2,21 | 17,7 | ± | 2,16 | 1,0 | ± | 0,28 |
|  | S | 17,8^b^ | ± | 1,87 | 76,7 | ± | 76,7 | 5,1 | ± | 1,06 | 0,0 | ± | 0,00 | 45,3^b^ | ± | 3,38 | 48,5 | ± | 2,57 | 6,2 | ± | 1,39 | 99,7^b^ | ± | 0,23 | 0,3 | ± | 0,23 | 0,0 | ± | 0,00 |
| Treatment | C | 10,4 | ± | 2,01 | 66,7 | ± | 2,59 | 22,8 | ± | 3,84 | 0,1 | ± | 0,10 | 33,2 | ± | 3,55 | 61,2 | ± | 3,30 | 5,6 | ± | 1,39 | 91,1 | ± | 2,29 | 8,4 | ± | 2,18 | 0,4 | ± | 0,20 |
|  | G | 11,4 | ± | 1,84 | 66,7 | ± | 2,37 | 21,6 | ± | 3,49 | 0,0 | ± | 0,00 | 33,7 | ± | 3,39 | 61,8 | ± | 3,03 | 4,5 | ± | 0,89 | 89,8 | ± | 2,27 | 9,6 | ± | 2,19 | 0,6 | ± | 0,23 |
| Breed * Treatment | FC | 2,5 | ± | 1,08 | 56,7 | ± | 3,28 | 40,6 | ± | 4,05 | 0,2 | ± | 0,21 | 21,0 | ± | 2,35 | 75,6 | ± | 1,94 | 3,3 | ± | 1,18 | 82,7 | ± | 3,46 | 16,5 | ± | 3,31 | 0,8 | ± | 0,37 |
|  | SC | 18,3 | ± | 2,69 | 76,7 | ± | 1,90 | 5,0 | ± | 1,52 | 0,0 | ± | 0,00 | 45,3 | ± | 5,20 | 46,8 | ± | 3,70 | 7,9 | ± | 2,43 | 99,6 | ± | 0,42 | 0,4 | ± | 0,42 | 0,0 | ± | 0,00 |
|  | FG | 5,4 | ± | 1,49 | 56,7 | ± | 2,51 | 37,9 | ± | 3,53 | 0,0 | ± | 0,00 | 22,0 | ± | 3,00 | 73,4 | ± | 2,61 | 4,6 | ± | 1,29 | 79,8 | ± | 2,82 | 19,0 | ± | 2,84 | 1,3 | ± | 0,42 |
|  | SG | 17,3 | ± | 2,67 | 76,7 | ± | 1,88 | 5,2 | ± | 1,52 | 0,0 | ± | 0,00 | 45,4 | ± | 4,50 | 50,2 | ± | 3,64 | 4,4 | ± | 1,28 | 99,8 | ± | 0,21 | 0,2 | ± | 0,21 | 0,0 | ± | 0,00 |
| P-values | Breed | < 0.001 | | | | | | | | | | | | < 0.001 | | | | | | | | | < 0.001 | | | | | | | | |
|  | Treatment | 0.36 | | | | | | | | | | | | 0.64 | | | | | | | | | 0.47 | | | | | | | | |
|  | Breed * Treatment | 0.30 | | | | | | | | | | | | 0.65 | | | | | | | | | 0.47 | | | | | | | | |

^a-b^ Per factor (breed, treatment or interaction), values in a column lacking a common superscript differ significantly (P < 0.05).

Supplementary Table S5b. Effects of breed, treatment and their interaction on broiler footpad dermatitis and hock burns across target weights (2.1 and 2.6 kg). Values are expressed as mean percentage of broilers having a specific score ± SE. For footpad dermatitis and hock burns, where score 0 = no lesion and 4 = severe lesion. F = fast-growing broilers; S = slower-growing broilers; C = Control; G = Gradient (n = 8 pens per breed * treatment combination).

|  |  | Footpad dermatitis | | | | | | | | | | | | Hock burns | | | | | | | | | | | | | | |
| --- | --- | --- | --- | --- | --- | --- | --- | --- | --- | --- | --- | --- | --- | --- | --- | --- | --- | --- | --- | --- | --- | --- | --- | --- | --- | --- | --- | --- |
|  |  | 0 |  | SE | 1 |  | SE | 2 |  | SE | 3 |  | SE | 0 |  | SE | 1 |  | SE | 2 |  | SE | 3 |  | SE | 4 |  | SE |
| Breed | F | 33,9^a^ | ± | 2,42 | 15,5 | ± | 1,20 | 50,3 | ± | 2,35 | 0,3 | ± | 0,17 | 92,2^a^ | ± | 1,17 | 6,9 | ± | 1,02 | 0,5 | ± | 0,22 | 0,4 | ± | 0,20 | 0,0 | ± | 0,00 |
|  | S | 81,7^b^ | ± | 2,01 | 7,2 | ± | 1,06 | 11,1 | ± | 1,57 | 0,0 | ± | 0,00 | 88,1^b^ | ± | 1,45 | 10,3 | ± | 1,29 | 0,1 | ± | 0,10 | 1,4 | ± | 0,47 | 0,1 | ± | 0,10 |
| Treatment | C | 60,5 | ± | 4,69 | 9,7 | ± | 1,19 | 29,7 | ± | 4,05 | 0,1 | ± | 0,10 | 90,7 | ± | 1,36 | 8,0 | ± | 1,13 | 0,3 | ± | 0,17 | 0,8 | ± | 0,40 | 0,1 | ± | 0,10 |
|  | G | 55,1 | ± | 4,93 | 13,0 | ± | 1,45 | 31,6 | ± | 4,04 | 0,2 | ± | 0,14 | 89,6 | ± | 1,37 | 9,2 | ± | 1,27 | 0,3 | ± | 0,17 | 0,9 | ± | 0,34 | 0,0 | ± | 0,00 |
| Breed * Treatment | FC | 37,5 | ± | 3,77 | 13,3 | ± | 1,58 | 49,0 | ± | 3,69 | 0,2 | ± | 0,21 | 92,7 | ± | 1,39 | 6,4 | ± | 1,19 | 0,4 | ± | 0,28 | 0,4 | ± | 0,28 | 0,0 | ± | 0,00 |
|  | SC | 83,5 | ± | 2,47 | 6,0 | ± | 1,26 | 10,5 | ± | 2,16 | 0,0 | ± | 0,00 | 88,7 | ± | 2,28 | 9,6 | ± | 1,87 | 0,2 | ± | 0,21 | 1,3 | ± | 0,74 | 0,2 | ± | 0,21 |
|  | FG | 30,3 | ± | 2,88 | 17,7 | ± | 1,69 | 51,5 | ± | 3,01 | 0,4 | ± | 0,28 | 91,7 | ± | 1,92 | 7,3 | ± | 1,70 | 0,6 | ± | 0,34 | 0,4 | ± | 0,28 | 0,0 | ± | 0,00 |
|  | SG | 79,9 | ± | 3,20 | 8,4 | ± | 1,70 | 11,7 | ± | 2,34 | 0,0 | ± | 0,00 | 87,5 | ± | 1,86 | 11,0 | ± | 1,82 | 0,0 | ± | 0,00 | 1,5 | ± | 0,61 | 0,0 | ± | 0,00 |
| P-values | Breed | < 0.001 | | | | | | | | | | | | 0.01 | | | | | | | | | | | | | | |
|  | Treatment | 0.32 | | | | | | | | | | | | 0.48 | | | | | | | | | | | | | | |
|  | Breed * Treatment | 0.98 | | | | | | | | | | | | 0.91 | | | | | | | | | | | | | | |

^a-b^ Per factor (breed, treatment or interaction), values in a column lacking a common superscript differ significantly (P < 0.05).

Supplementary Table S6. Effects of breed, treatment and their interaction on friability and wetness across target weights (0.3, 0.8, 1.3 and 2.4 kg). Values are expressed as mean percentage of broiler pens having a specific score ± SE. Where score 1 = low litter quality (completely caked or very wet) and 10 = high litter quality (completely friable or dry). F = fast-growing broilers; S = slower-growing broilers; C = Control; G = Gradient (n = 8 pens per breed * treatment combination).

|  |  | Friability | | | | | | | | | | | | | | | | | | | | | Wetness | | | | | | | | | | | |
| --- | --- | --- | --- | --- | --- | --- | --- | --- | --- | --- | --- | --- | --- | --- | --- | --- | --- | --- | --- | --- | --- | --- | --- | --- | --- | --- | --- | --- | --- | --- | --- | --- | --- | --- |
|  |  | 4 |  | SE | 5 |  | SE | 6 |  | SE | 7 |  | SE | 8 |  | SE | 9 |  | SE | 10 |  | SE | 6 |  | SE | 7 |  | SE | 8 |  | SE | 10 |  | SE |
| Breed | F | 1,6^a^ | ± | 1,10 | 16,4 | ± | 4,46 | 19,5 | ± | 4,93 | 14,8 | ± | 4,27 | 21,1 | ± | 5,08 | 1,6 | ± | 1,56 | 25,0 | ± | 5,46 | 49,2 | ± | 6,25 | 25,8 | ± | 5,45 | 0,0 | ± | 0,00 | 25,0 | ± | 5,46 |
|  | S | 0,0^b^ | ± | 0,00 | 1,6 | ± | 1,10 | 4,7 | ± | 1,84 | 17,2 | ± | 4,05 | 12,5 | ± | 3,15 | 39,1 | ± | 5,84 | 25,0 | ± | 5,46 | 28,1 | ± | 5,66 | 9,4 | ± | 3,67 | 37,5 | ± | 6,10 | 25,0 | ± | 5,46 |
| Treatment | C | 1,6 | ± | 1,10 | 7,8 | ± | 2,99 | 12,5 | ± | 3,86 | 14,1 | ± | 3,77 | 16,4 | ± | 4,17 | 22,7 | ± | 5,09 | 25,0 | ± | 5,46 | 36,7 | ± | 6,02 | 19,5 | ± | 4,93 | 18,8 | ± | 4,92 | 25,0 | ± | 5,46 |
|  | G | 0,0 | ± | 0,00 | 10,2 | ± | 3,72 | 11,7 | ± | 3,82 | 18,0 | ± | 4,51 | 17,2 | ± | 4,34 | 18,0 | ± | 4,64 | 25,0 | ± | 5,46 | 40,6 | ± | 6,19 | 15,6 | ± | 4,57 | 18,8 | ± | 4,92 | 25,0 | ± | 5,46 |
| Breed * Treatment | FC | 3,1 | ± | 2,17 | 14,1 | ± | 5,61 | 20,3 | ± | 7,05 | 10,9 | ± | 4,88 | 23,4 | ± | 7,44 | 3,1 | ± | 3,13 | 25,0 | ± | 7,78 | 45,3 | ± | 8,80 | 29,7 | ± | 8,05 | 0,0 | ± | 0,00 | 25,0 | ± | 7,78 |
|  | SC | 0,0 | ± | 0,00 | 1,6 | ± | 1,56 | 4,7 | ± | 2,62 | 17,2 | ± | 5,77 | 9,4 | ± | 3,51 | 42,2 | ± | 8,43 | 25,0 | ± | 7,78 | 28,1 | ± | 8,08 | 9,4 | ± | 5,24 | 37,5 | ± | 8,70 | 25,0 | ± | 7,78 |
|  | FG | 0,0 | ± | 0,00 | 18,8 | ± | 7,01 | 18,8 | ± | 7,01 | 18,8 | ± | 7,01 | 18,8 | ± | 7,01 | 0,0 | ± | 0,00 | 25,0 | ± | 7,78 | 53,1 | ± | 8,96 | 21,9 | ± | 7,42 | 0,0 | ± | 0,00 | 25,0 | ± | 7,78 |
|  | SG | 0,0 | ± | 0,00 | 1,6 | ± | 1,56 | 4,7 | ± | 2,62 | 17,2 | ± | 5,77 | 15,6 | ± | 5,24 | 35,9 | ± | 8,17 | 25,0 | ± | 7,78 | 28,1 | ± | 8,08 | 9,4 | ± | 5,24 | 37,5 | ± | 8,70 | 25,0 | ± | 7,78 |
| P-values | Breed | < 0.001 | | | | | | | | | | | | | | | | | | | | | NA | | | | | | | | | | | |
|  | Treatment | 0.40 | | | | | | | | | | | | | | | | | | | | | NA | | | | | | | | | | | |
|  | Breed * Treatment | 0.85 | | | | | | | | | | | | | | | | | | | | | NA | | | | | | | | | | | |

^a-b^ Per factor (breed, treatment or interaction), values in a column lacking a common superscript differ significantly (P < 0.05).

Supplementary Figure S1. A, B and C) Boxplots and D, E and F) Means for activity (pixel changes) of F = fast-growing broilers and S = slower-growing broilers for the dim (blue) or bright (red) area of the gradient lighting and for the control (green) lighting per week of age. A and D) central zone (control = 20 lux, bright = 97 lux and dim = 57 lux), B and E) intermediate zone (control = 20 lux, bright = 97-204 lux and dim = 24-57 lux), C and F), wall zone (control = 20 lux, bright = 204 lux and dim = 24 lux). Solid lines represent the different pens (note that replicates per breed and age differ).

Supplementary Figure S2. A, B and C) Boxplots and D, E and F) Means for distribution (# chickens) of F = fast-growing broilers and S = slower-growing broilers for the dim (blue) or bright (red) area of the gradient lighting and for the control (green) lighting per week of age. A and D) central zone (control = 20 lux, bright = 97 lux and dim = 57 lux), B and E) intermediate zone (control = 20 lux, bright = 97-204 lux and dim = 24-57 lux), C and F), wall zone (control = 20 lux, bright = 204 lux and dim = 24 lux). Solid lines represent the different pens (note that replicates per breed and age differ).
